# Reliability of Intra-Retinal Layer Thickness Estimates

**DOI:** 10.1371/journal.pone.0137316

**Published:** 2015-09-08

**Authors:** Timm Oberwahrenbrock, Maria Weinhold, Janine Mikolajczak, Hanna Zimmermann, Friedemann Paul, Ingeborg Beckers, Alexander U. Brandt

**Affiliations:** 1 NeuroCure Clinical Research Center, Charité–Universitätsmedizin Berlin, Berlin, Germany; 2 Optics and Laser Technology Laboratory, Beuth University of Applied Sciences, Berlin, Germany; 3 Department of Neurology, Charité–Universitätsmedizin, Berlin, Germany; Friedrich-Alexander University Erlangen, GERMANY

## Abstract

**Purpose:**

Measurement of intra-retinal layer thickness using optical coherence tomography (OCT) has become increasingly prominent in multiple sclerosis (MS) research. Nevertheless, the approaches used for determining the mean layer thicknesses vary greatly. Insufficient data exist on the reliability of different thickness estimates, which is crucial for their application in clinical studies. This study addresses this lack by evaluating the repeatability of different thickness estimates.

**Methods:**

Studies that used intra-retinal layer segmentation of macular OCT scans in patients with MS were retrieved from PubMed. To investigate the repeatability of previously applied layer estimation approaches, we generated datasets of repeating measurements of 15 healthy subjects and 13 multiple sclerosis patients using two OCT devices (Cirrus HD-OCT and Spectralis SD-OCT). We calculated each thickness estimate in each repeated session and analyzed repeatability using intra-class correlation coefficients and coefficients of repeatability.

**Results:**

We identified 27 articles, eleven of them used the Spectralis SD-OCT, nine Cirrus HD-OCT, two studies used both devices and two studies applied RTVue-100. Topcon OCT-1000, Stratus OCT and a research device were used in one study each. In the studies that used the Spectralis, ten different thickness estimates were identified, while thickness estimates of the Cirrus OCT were based on two different scan settings. In the simulation dataset, thickness estimates averaging larger areas showed an excellent repeatability for all retinal layers except the outer plexiform layer (OPL).

**Conclusions:**

Given the good reliability, the thickness estimate of the 6mm-diameter area around the fovea should be favored when OCT is used in clinical research. Assessment of the OPL was weak in general and needs further investigation before OPL thickness can be used as a reliable parameter.

## Introduction

Optical coherence tomography (OCT) is a widely established means of acquiring high-resolution retinal images [1]. The technique is based on low coherence interferometry, in which near-infrared light is used to generate spatial images of biological target tissue. Over the years, OCT evolved into a number of different approaches. A significant breakthrough in development was the transition from *time domain* OCT (TD-OCT), which is hampered in terms of its application in medicine by low acquisition speed and limited resolution, to *frequency*- (or *spectral*-) *domain* OCT (SD-OCT), which greatly increased imaging speed while simultaneously improving the signal-to-noise ratio [2].

Today’s SD-OCT devices allow high-resolution, 3D imaging of retinal tissue. They expand the use of OCT from macroscopic qualitative interpretation towards quantitative applications. One emerging field for this quantitative OCT is the measurement of neurodegeneration and neuroinflammation in neurological disorders. As part of the central nervous system, the retina is an excellent site for monitoring neurological pathologies, from well-known and economically significant diseases like multiple sclerosis (MS) [3], Parkinson’s disease [4] or Alzheimer’s disease [5], to less common diseases like neuromyelitis optica [6], Friedreich’s ataxia [7], Susac syndrome [8] or the spinocerebellar ataxias [9].

Recently, quantitative OCT imaging of the retina has shifted from the peripapillary retinal nerve fiber layer (RNFL) thickness as a marker of retinal neurodegeneration to distinct macular intra-retinal layer thicknesses. However, quantification of intra-retinal layer thickness requires the segmentation of OCT scans into the respective layers. Currently, this task is performed either manually, by experienced graders, semi-automatically, with manual inspectors correcting automated results, or fully automatically. Moreover, the algorithms used to automate the segmentation have been developed independently by the device manufacturers. Consequently, several post-processing algorithms exist that aim to meet this requirement as built-in or add-on software, but the lack of standardization between these limits their prospects for diagnosis and disease monitoring. Until this lack is redressed, OCT, promising as it may be, cannot be used as a reliable marker for neurodegeneration in either therapeutic trials or clinical care, as previously suggested [10].

The necessary reliability standards can be met if two current deficiencies are redressed: Firstly, the current algorithms have to be improved to ensure that all techniques discriminate reliably between different layers [11]. Secondly, the approaches used to calculate the final outcome values for the mean layer thickness differ greatly in area and amount of single measurements included for averaging. These *layer thickness estimates* need to be standardized and investigated for reliability and comparability [12]. Especially when semi-automatic segmentation is performed, which is standard practice today, researchers tend towards smaller thickness estimates with a limited number of included B-scans in order to decrease workload for manual inspection. The goal of the present study was to investigate the precision of different layer thickness estimates for intra-retinal OCT segmentation measurements under repeatability conditions. We were especially interested in studies in MS, since here the most studies using quantitative OCT have been published.

## Methods

### Literature Review

All research articles that included intra-retinal segmentation of macular OCT scans in MS were retrieved from the PubMed database using the following search term: “optical coherence tomography multiple sclerosis” (search performed on 01/24/2014). Only articles written in English that discussed thickness or volume estimates of distinct retinal layers or combined layers were included. Exclusion criteria were the use of retinal segmentation in OCT scans other than macular scans (i.e. scans of the optic nerve head) and if the segmentation was solely performed to determine the thickness of the whole retina (from inner limiting membrane to Bruch’s membrane or retinal pigment epithelium).

### Subjects

To test the repeatability of different layer thickness estimates under various conditions we generated three datasets: two control cohorts of 15 healthy subjects each underwent three consecutive examinations with Spectralis SD-OCT (measured by MW) or Cirrus HD-OCT (measured by TO) and 13 MS/CIS patients receiving two consecutive examinations with both OCT devices (measured by JM). A detailed demographic overview of the investigated cohorts can be found in Table A in S1 File. Subject inclusion criteria were best corrected visual acuity of at least 0.8 according to ETDRS charts, using the Optec 6500P measurement device (Stereo Optical Inc., Chicago, Illinois, USA). Exclusion criteria were any history of neurological (except MS) or ophthalmological diseases and a refractive error of more than ±5.0 diopters. None of the healthy subjects showed any retinal alterations that affected OCT imaging. Patients were not matched to controls but were chosen because of known retinal affections related to MS or ON in case of CIS patients. All participants gave informed, written consent.

### Ethics

The study was approved by the ethics committee of the Charité–Universitätsmedizin Berlin and was conducted in accordance with the Declaration of Helsinki in its current version and applicable German laws.

### Optical Coherence Tomography

**Fig 1:**
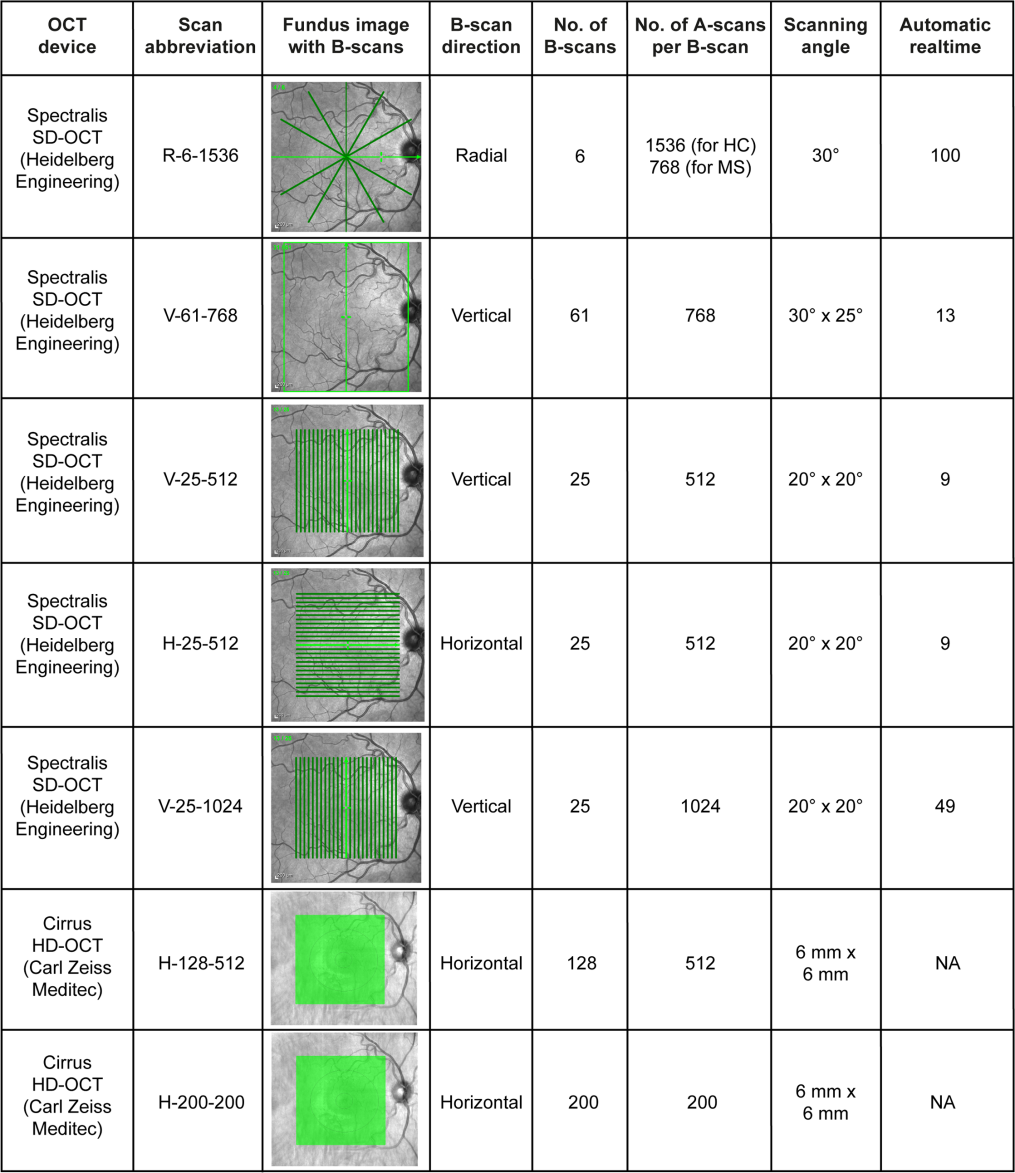
OCT scan settings used for simulation of the repeatability of different thickness estimates. The first column indicates the OCT device used to produce the scan in each row. The following column names each set of scanning parameter as a concatenation of scan direction, the number of B-scans and the number of A-scans (see columns 4–6). The third column shows for each scan design the generated OCT scans as lines or boxes on exemplary fundus images. The heights and widths of the scan areas are given in column 7, followed by the number of averaging frames in automatic real-time mode in the last column (only applicable for the Spectralis SD-OCT scan settings). NA = not applicable.

OCT examinations were performed with the Spectralis SD-OCT (Heidelberg Engineering, Heidelberg, Germany; Heidelberg eye explorer version 5.7.5.0) and the Cirrus HD-OCT 4000 (Carl Zeiss Meditec, Dublin, USA; instrument software version 6.5.0.772). Both eyes of every subject were measured and included in the analysis. We determined five different Spectralis scan designs replicating the settings used in the published studies that met the inclusion criteria (Fig 1). Previous studies with Cirrus OCT made use of the two standardly implemented macula volume scans (Fig 1).

For our repeatability datasets all scan protocols were applied with at least 5 minutes between consecutive examinations. The test subjects were asked to leave the room between examinations and the OCT devices were reset to an initial position. All measurements were performed with activated eye tracker, but without the device’s follow-up function.

### Intra-Retinal Segmentation

**Fig 2:**
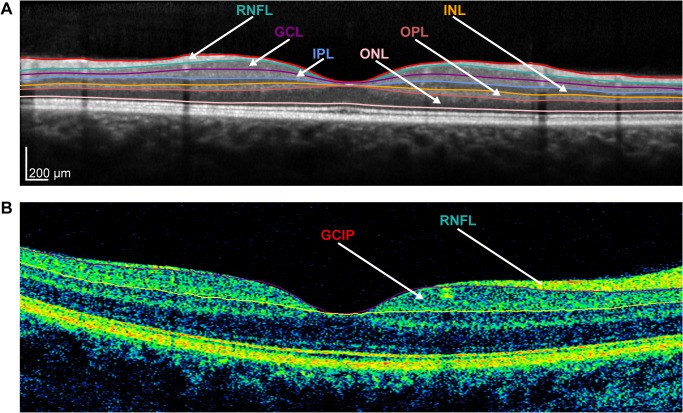
Intra-retinal layer segmentation of OCT B-scans. (A) Exemplary B-scan of a Spectralis SD-OCT with manually reviewed and corrected segmentation into the following retinal layers: retinal nerve fiber layer (RNFL), ganglion cell layer (GCL), inner plexiform layer (IPL), inner nuclear layer (INL), outer plexiform layer (OPL) and outer nuclear layer (ONL). (B) Exemplary Cirrus HD-OCT B-scan with segmentation in the RNFL and combined ganglion cell and inner plexiform (GCIP) layer.

**Fig 3:**
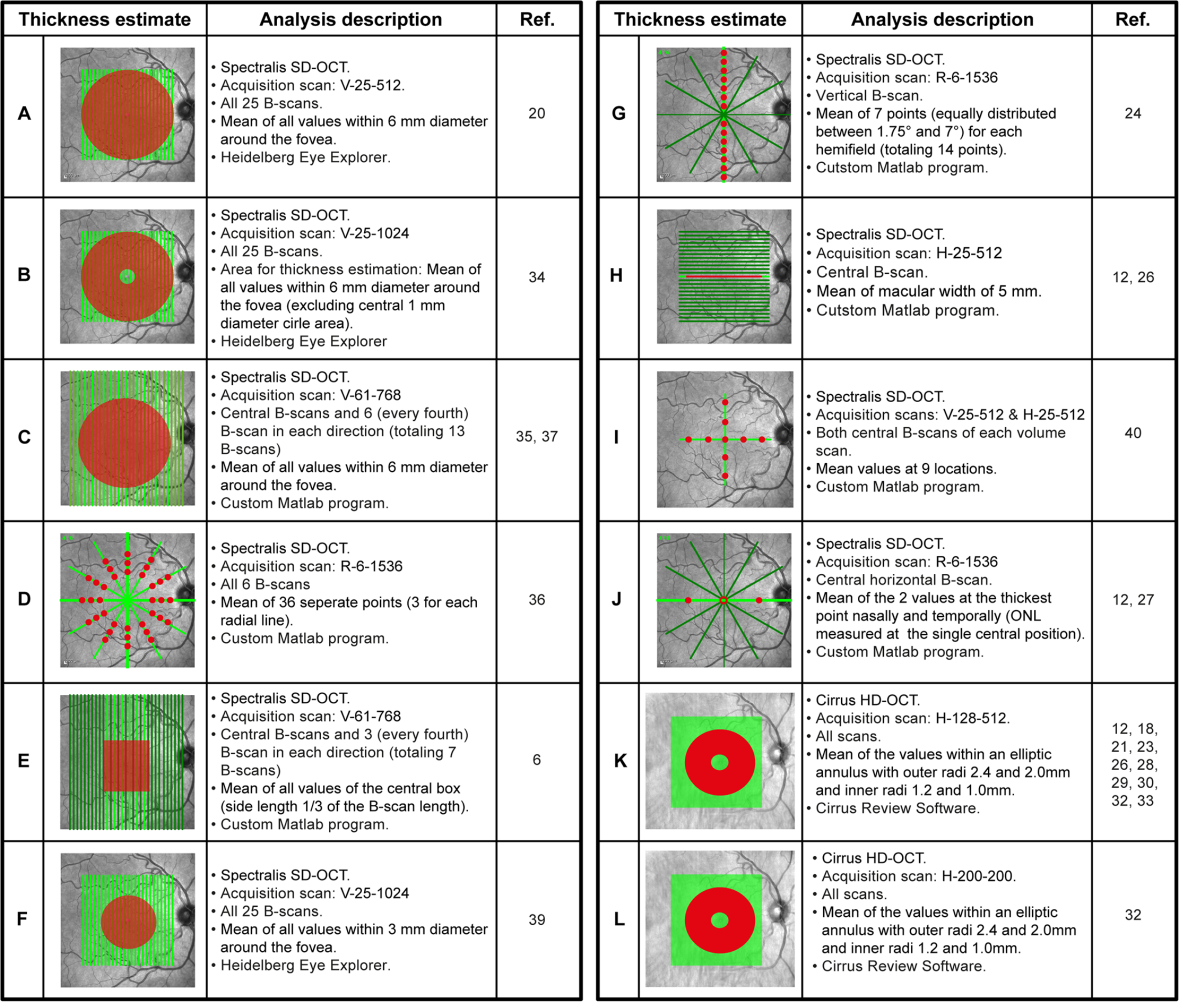
Explanation of different thickness estimates used for the simulation of repeatability. The red areas or points on the fundus images indicate the values that were averaged to generate the layer thickness estimates. The text to the right of each image refers to (top-to-bottom): 1) the used OCT device 2) the applied scan as elucidated in Fig 1; 3) the (subset of) B-scans used for the thickness estimate; 4) the procedure used to calculate the thickness estimates; 5) the program used for calculating the thickness estimate. The last column indicated the article(s) in which the layer estimated was applied. For a detailed overview of the segmentation methods applied in the retrieved articles, see Table B in S1 File. Note: the number and spacing of B-scans on the fundus image for the thickness estimates C and E are not correct due to space limitations.

To analyze repeatability of different Spectralis layer thickness estimates, initial intra-retinal segmentation was performed using the Heidelberg Eye Explorer software (Version 6.0.0.2), after which an operator (MW for controls and TO for patients) reviewed the results and occasionally corrected the segmentation lines where necessary (semi-automatic segmentation). Segmentation line correction was performed individually for each scan without using any other scan for reference. Fig 2A shows an exemplary B-scan from a Spectralis SD-OCT including the segmentation lines used to delineate the following retinal layers: RNFL, ganglion cell layer (GCL), inner plexiform layer (IPL), inner nuclear layer (INL), outer plexiform layer (OPL) and outer nuclear layer (ONL). As previous works have utilized the combined ganglion cell and inner plexiform layers (GCIP), we also included this parameter in our analysis. The positions of the segmentation lines were then exported to a spreadsheet, which was imported into Matlab (Version 2012b, MathWorks, Munich, Germany) to calculate mean layer thicknesses with different thickness estimates (Fig 3, thickness estimates A—J). For thickness estimates based on a single B-scan only, we extracted the corresponding B-scans from an appropriate volume scan (Fig 3, thickness estimates G—J).

The Cirrus volume scans were automatically segmented with the Cirrus review software (6.5.0.772) with no corrections of segmentation lines possible in this software. The mean thicknesses were calculated for the RNFL and the GCIP (see Fig 2B) and exported to XML files for analysis. Layer estimates for Cirrus scans (Fig 3, thickness estimates K and L) were estimated in an elliptical annulus around the fovea with outer radii of 2.4 mm (horizontal) and 2.0 mm (vertical), and inner radii of 0.6 mm (horizontal) and 0.5 mm (vertical), respectively [13].

### Statistical Analysis

The thickness estimates for each layer were analyzed using modified Bland-Altman plots [14]. Here, the differences between each pair of repeated measurements were plotted against the mean of the pair. Plots were visually inspected for the occurrence of systematic bias or outliers. Next, the reliability of repeated measurements was calculated as intra-class correlation coefficients (ICC) based on the variance components of a one-way ANOVA fixed effects model with single measures (also referred to as ICC(1,1)) in R (Version 3.1.3) with the package ICC [15]. The ICC can take values from 0 to 1, with 1 representing perfect correlation and 0 no correlation. We considered ICC values above 0.9 as excellent, ICC values between 0.9 and 0.8 as moderate and less than 0.8 as insufficient, as previously suggested [16]. ICC statistics were performed for the mixed cohort of MS/CIS patients and healthy controls, as well as for each group separately. Since ICC are relative indices that are influenced by the heterogeneity of the measurement across different subjects, we also analyzed the coefficient of repeatability (CR) as an absolute reliability index, which is calculated by multiplying the within-subject standard deviation with 2.77 (= √2 x 1.96). This yields the interval of the absolute difference between repeated measurements with a probability of 95% [16]. The CRs propose a magnitude for clinically relevant changes in retinal layer mean thicknesses.

## Results

### Published MS Studies with Intra-Retinal Segmentation of Macular OCT Scans

The search term “optical coherence tomography multiple sclerosis” yielded 279 articles in PubMed, of which 27 met the inclusion criteria [6,12,17–41]. Of the latter, eleven applied Spectralis SD-OCT (Heidelberg Engineering, Heidelberg, Germany) [6,20,24,27,34–37,39–41]; nine used Cirrus OCT (Carl Zeiss Meditec, Dublin, CA, USA) [18,21,23,25,28–30,32,33]; two studies used both devices [12,26] and two used RTVue-100 (Optovue, Fremont, CA, USA) [19,38]. The OCT-1000 (Topcon Corp., Itabashi, Japan) [31], the Stratus OCT (Carl Zeiss Meditec, Dublin, CA, USA) [22] and a custom research device [17] were used in one study each. An analysis was performed of the OCT examinations used in each study, as reflected by the scan settings listed in Table B in S1 File and a detailed overview of the thickness estimates, the segmentation software type (automatic, semi-automatic or manual) and the investigated retinal layers in Table C in S1 File.

For Cirrus OCT two different thickness estimates were used and for both automatic segmentation software were applied (Table C in S1 File). In contrast, we identified ten different thickness estimates used for Spectralis OCT scans. In this study, we analyzed the repeatability of Spectralis and Cirrus OCT layer thickness estimates by generating repetitive OCT measurements in MS/CIS patients and healthy controls. Fig 3 summarizes the thickness estimates, how we implemented them to investigate the repeatability and in which studies they were applied.

### Repeatability of OCT Layer Thickness Estimates

The intra-observer repeatability of the twelve OCT layer thickness estimates (Fig 3) and for each of the seven retinal layers (including the composite GCIP layer) are presented for the mixed cohort of patients and controls by ICC statistics in Table 1 and CR in Table 2. In summary, thickness estimates averaging larger areas (Spectralis thickness estimates A–E and estimates K and L for Cirrus) showed mostly excellent ICC values (>0.9) and low CR (1–4 μm). The deviation of thickness estimates F–J were broader across the retinal layers and some only reached moderate or even insufficient ICC levels. Particularly thickness estimate J was characterized by higher degree of measurement noise (CR >8 μm for all layers).

**Table 1:** Repeatability of thickness estimates and retinal layers in a mixed cohort of healthy controls and MS/CIS patients as measured by intraclass correlation coefficients (ICC) with 95%-confidence intervals.

Estimate	mRNFL	GCL	IPL	GCIP	INL	OPL	ONL
**A**	0.98 [0.99–0.97]	0.99 [0.99–0.98]	0.99 [0.99–0.98]	1.00 [1.00–1.00]	0.97 [0.98–0.96]	0.82 [0.88–0.72]	0.98 [0.99–0.97]
**B**	0.97 [0.98–0.95]	0.99 [1.00–0.99]	0.99 [0.99–0.98]	1.00 [1.00–1.00]	0.96 [0.98–0.94]	0.75 [0.84–0.63]	0.97 [0.98–0.95]
**C**	0.98 [0.99–0.97]	1.00 [1.00–0.99]	0.99 [1.00–0.99]	1.00 [1.00–1.00]	0.98 [0.99–0.96]	0.78 [0.86–0.68]	1.00 [1.00–0.99]
**D**	0.98 [0.99–0.97]	0.96 [0.98–0.94]	0.96 [0.97–0.93]	0.98 [0.99–0.97]	0.87 [0.92–0.80]	0.87 [0.92–0.80]	1.00 [1.00–0.99]
**E**	0.97 [0.98–0.95]	0.99 [1.00–0.99]	0.98 [0.99–0.97]	1.00 [1.00–0.99]	0.90 [0.94–0.85]	0.69 [0.80–0.56]	0.99 [1.00–0.99]
**F**	0.88 [0.92–0.81]	0.99 [0.99–0.99]	0.98 [0.99–0.97]	0.99 [1.00–0.99]	0.83 [0.89–0.75]	0.62 [0.75–0.47]	0.92 [0.95–0.87]
**G**	0.96 [0.98–0.94]	0.93 [0.95–0.88]	0.89 [0.93–0.83]	0.94 [0.96–0.91]	0.72 [0.82–0.59]	0.68 [0.79–0.54]	0.99 [0.99–0.99]
**H**	0.92 [0.95–0.87]	0.96 [0.98–0.94]	0.96 [0.97–0.93]	0.98 [0.99–0.97]	0.73 [0.82–0.60]	0.59 [0.72–0.43]	0.98 [0.99–0.98]
**I**	0.81 [0.88–0.71]	0.96 [0.97–0.93]	0.93 [0.96–0.89]	0.98 [0.99–0.97]	0.83 [0.90–0.75]	0.55 [0.69–0.38]	0.98 [0.99–0.97]
**J**	0.92 [0.95–0.88]	0.82 [0.89–0.73]	0.77 [0.86–0.66]	0.86 [0.91–0.79]	0.76 [0.85–0.65]	0.80 [0.87–0.70]	0.92 [0.95–0.87]
**K**	0.98 [0.99–0.97]	NA	NA	1.00 [1.00–1.00]	NA	NA	NA
**L**	0.99 [0.99–0.98]	NA	NA	1.00 [1.00–1.00]	NA	NA	NA

**Abbreviations: mRNFL** = macular retinal nerve fiber layer, **GCL** = ganglion cell layer, **IPL** = inner plexiform layer, **GCIP** = combined GCL and IPL, INL = inner nuclear layer, **OPL** = outer plexiform layer, **ONL** = outer nuclear layer.

**Table 2:** Coefficient of repeatability (CR, in μm) of thickness estimates and retinal layers in a mixed cohort of healthy controls and MS/CIS patients.

Estimate	mRNFL	GCL	IPL	GCIP	INL	OPL	ONL
**A**	2.06	1.50	1.18	1.23	1.15	2.31	2.30
**B**	2.91	1.23	1.10	1.22	1.31	2.91	3.21
**C**	2.24	1.02	0.85	0.88	1.06	2.57	2.50
**D**	3.23	1.87	1.80	2.33	2.20	1.82	2.06
**E**	2.43	1.47	1.62	1.70	2.31	3.75	3.41
**F**	2.87	2.40	2.14	2.90	3.48	5.89	6.28
**G**	4.35	2.92	2.68	4.40	3.77	2.99	3.44
**H**	3.64	3.03	2.31	3.67	4.50	5.40	5.15
**I**	3.78	3.75	3.53	4.34	4.55	6.00	5.56
**J**	9.07	9.19	8.64	13.23	8.88	9.90	17.75
**K**	1.91	NA	NA	1.66	NA	NA	NA
**L**	1.60	NA	NA	1.38	NA	NA	NA

**Abbreviations: mRNFL** = macular retinal nerve fiber layer, **GCL** = ganglion cell layer, **IPL** = inner plexiform layer, **GCIP** = combined GCL and IPL, INL = inner nuclear layer, **OPL** = outer plexiform layer, **ONL** = outer nuclear layer.

The widths of the ICC confidence intervals (CI) as a measure of the precision were highly dependent on the ICC values: with high ICC values (>0.90) we also found very narrow CI widths (<0.1), but CI widths were wider for lower ICC values.

**Fig 4:**
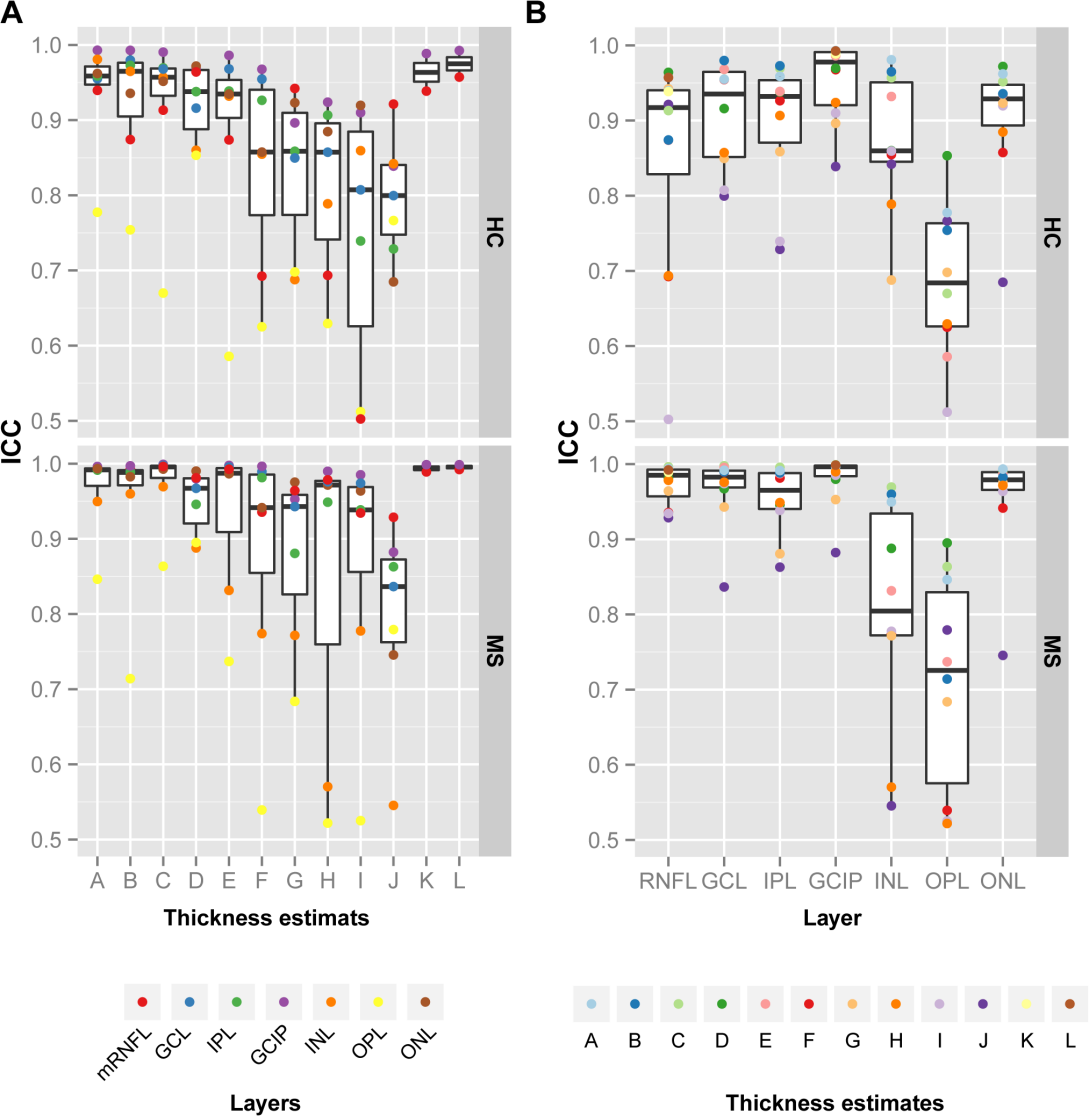
Intraclass correlation coefficients (ICC) for different thickness estimates and retinal layers in MS/CIS patients and healthy controls. Intraclass correlation coefficient (ICC) for the distinct cohorts of healthy controls (HC) and patients (MS) ordered for (A) the twelve thickness estimates (Fig 3) and (B) the seven retinal layers. Abbreviations: mRNFL = macular retinal nerve fiber layer, GCL = ganglion cell layer, IPL = inner plexiform layer, GICP = combined GCL and IPL, INL = inner nuclear layer, OPL = outer plexiform layer, ONL = outer nuclear layer.

**Fig 5:**
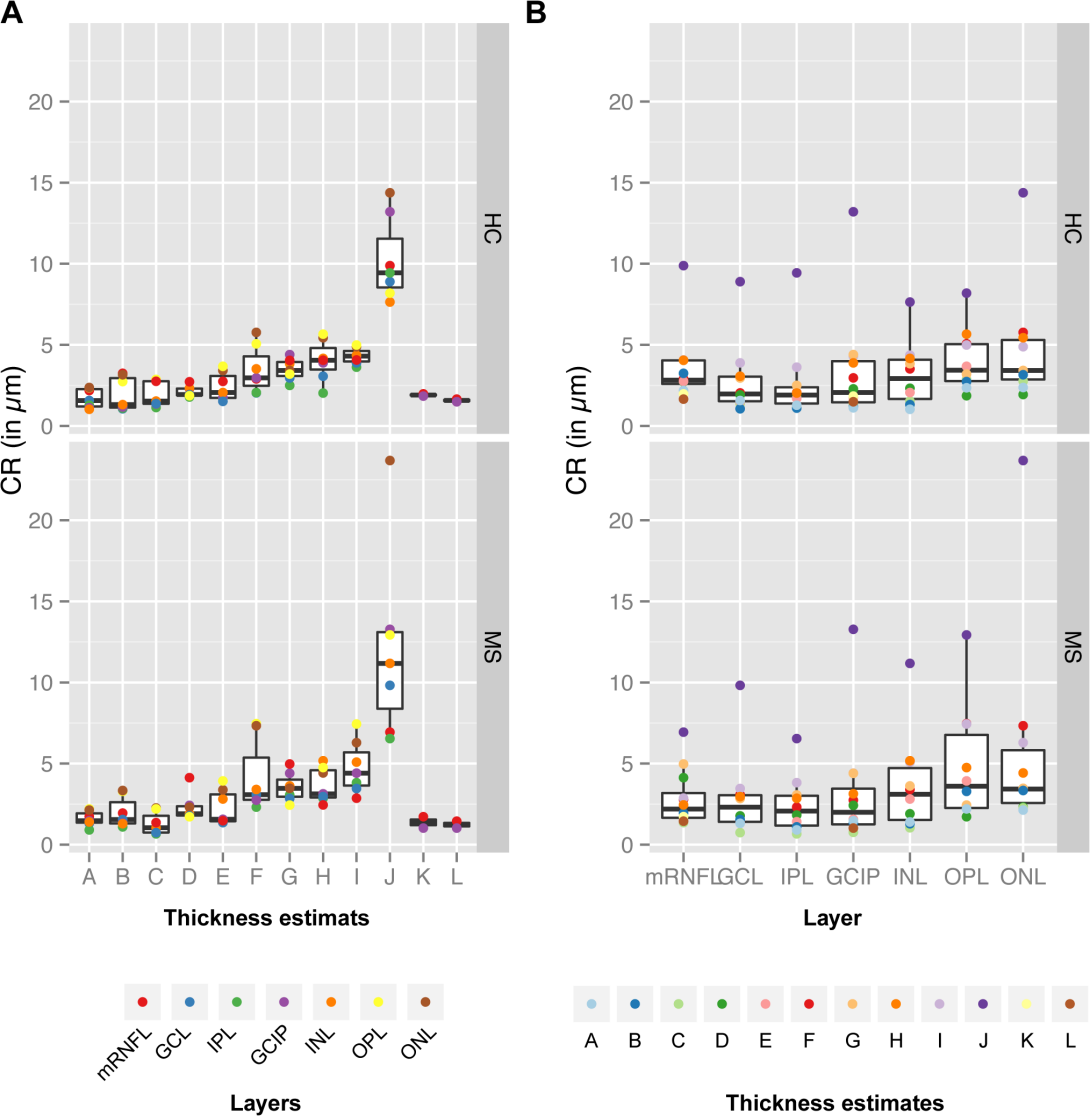
Coefficient of repeatability (CR) for different thickness estimates and retinal layers in MS/CIS patients and healthy controls. Coefficient of repeatability (CR) for the distinct cohorts of healthy controls (HC) and patients (MS) ordered for (A) the 12 thickness estimates (Fig 3) and (B) the 7 retinal layers. **Abbreviations**: **mRNFL** = macular retinal nerve fiber layer, **GCL** = ganglion cell layer, **IPL** = inner plexiform layer, **GICP** = combined GCL and IPL, **INL** = inner nuclear layer, **OPL** = outer plexiform layer, **ONL** = outer nuclear layer.

In addition to the mixed cohort, were performed distinct analysis of the patient and control cohorts, see Fig 4 for ICC statistics and Fig 5 for CR measures. The comparison of the repeatability results for the different thickness estimates (Fig 4A) revealed a similar distribution for both cohorts. Of note is, that both cohorts were measured, reviewed and manually corrected by different operators, which might introduce additional bias to the group comparison. Nevertheless, the distribution of ICC values indicate that our previous findings are valid independent of the health status of the measured retina.

### Repeatability of Different Retinal Layers

**Fig 6:**
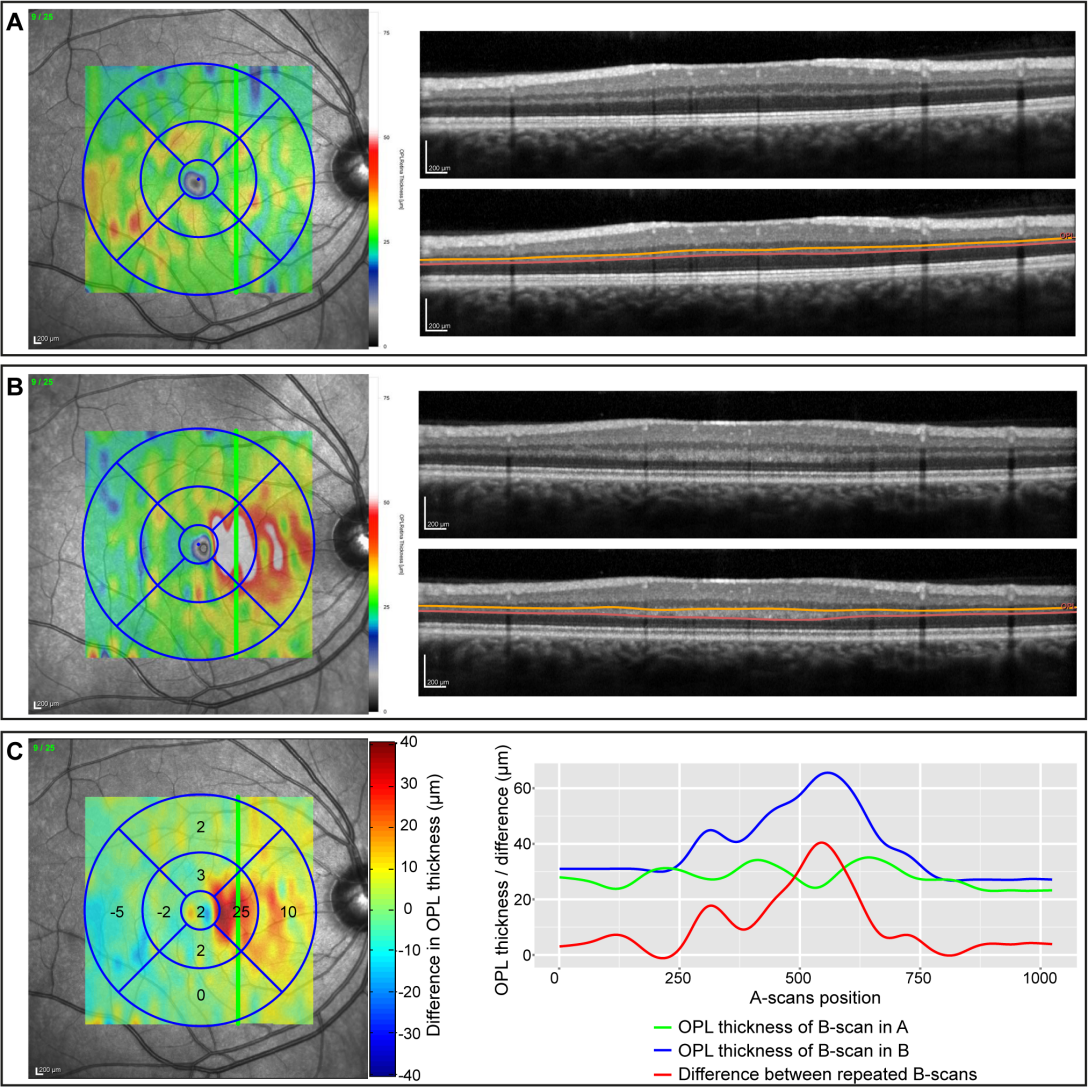
Differences in the outer plexiform layer (OPL) in repeated OCT measurements. (A) and (B) show two repeated OCT measurements (16 min between both examinations) of the same subject with the same scan settings (Scan V-25-1024 in Fig 1). On the left, the fundus images are given with an overlay of the OPL thickness maps generated by the Eye Explorer software (Heidelberg Engineering, Germany). The B-scans (with and without OPL segmentation lines) are shown on the right and are located at the position indicated by the green line on the fundus images. (C) The difference map on the left was calculated by subtracting the OPL thickness map in (A) from the map in (B) (custom Matlab program). The values in the grid are the mean OPL thickness differences for each sector. The right graph maps the OPL thickness of the B-scans in (A) (green line) and (B) (blue line), respectively. The red line indicates the difference between the repeated B-scans.

To further investigate differences in single retinal layers, Fig 4B and Fig 5B show the repeatability indices against the distinct layers. The weakest correlation between repeated measurements was present in the OPL in which thickness estimates mainly had lower ICC values indicative for insufficient repeatability. This high measurement noise can be explained by the divergent appearance of the OPL in some of the repeated measurements: Fig 6 shows an example of the OPL in two repeated measurements of the same subject taken 16 min apart (Fig 6A and 6B). In this case, the averaged local OPL differences between both examinations reached up to 25 μm and on single B-scans the differences were up to 40 μm (see Fig 6C).

### Systematic Error Analysis

Visual review of the Bland-Altman plots for individual thickness estimates and retinal layers showed no systematic errors or outliers (data not shown).

## Discussion

Our investigation showed that a wide range of segmentation approaches and layer thickness estimates are currently used in intra-retinal segmentation studies in MS, unfortunately, with equally divergent reliability. As a result, readers should be careful when interpreting published studies using weak thickness estimates, identified by this study.

Spectralis SD-OCT and Cirrus HD-OCT are currently the two most common devices used for intra-retinal segmentation in MS. For the Cirrus HD-OCT the segmentation algorithm of the GCIP has been an integral part of the software since version 6.0 and its reproducibility had been investigated elsewhere [13]. Our repeatability analysis for both of the two standard Cirrus scanning protocols showed very high intra-observer reliability. While all but one of the studies using the Cirrus rely on this built-in automatic segmentation, we identified a total of ten different thickness estimates that were used in studies with the Spectralis device. Our analysis of the different Spectralis thickness estimates revealed that estimates averaging larger areas (thickness estimates A–E, Fig 3) showed excellent repeatability for all retinal layers except the OPL. In contrast, thickness estimates based on only a few single scan locations are more strongly affected by measurement noise introduced in repeated examinations and thus show less reliability. Of note is that the three thickness estimates (A—C) using the 6mm-diameter area around the fovea with different scan settings performed similarly well, independently of the transversal resolution and image averaging rate (automatic real-time, ART). Although higher scan settings, which also result in slower scanning speed, might not be beneficial in terms of repeatability, they can have an impact on the sensitivity. In this study, we did not assess the sensitivity of thickness estimates to detect changes in individual layer measurements, but instead only investigated the repeated measurement reliability of different thickness estimates. However, low CR in our study might indicate that layer measurements are potentially sensitive for tracking longitudinal changes, but this needs to be investigated further.

Studies in MS patients have revealed pronounced reduction in the ganglion cell layer and this may be the most important target for tracking neurodegeneration at least in MS patients [37,39]. Since the ganglion cell layer is thickest in the macula, thickness estimates that entirely cover the macula likely have the best sensitivity for tracking changes. The CR of the GCL (1.02 μm), IPL (0.85 μm) and the CR of the GCIP (0.88 μm) using the thickness estimate C had the overall best performance. A recent study estimated yearly GCL/GCIP loss at 0.5 μm using layer estimate K, suggesting that SD-OCT should be able to track changes over 24 months [42]. After acute optic neuritis, average GCIP loss was reported at 9 μm in a recent study [23], making several thickness estimate good candidates to track GCL/GCIP changes after acute ON.

Another layer of interest is the INL, in which thickening in the area of the macula has been reported in relation to optic neuritis and MS disease activity [28,35,36,43]. Here, the CR was just above 1 μm in layer estimate A—C. After acute optic neuritis, intermittent INL thickening was reported by two studies in the range of 1–4 μm [23,34]. There is currently no estimate for the magnitude of annual changes in MS outside acute ON. Considering the above CR, current SD-OCT might just fall short in reliably detecting INL changes over typical observation times both in acute ON and in MS.

The OPL results deserve special mention. Axons in the OPL of photoreceptor nuclei, commonly known as Henle's fiber layer (HFL), run obliquely in the tissue and its OCT appearance is highly dependent on the angle of the OCT’s light beam relative to this layer [44]. The HFL can either appear hyperintense or hypointense, which can skew the determination of the OPL thickness (Fig 6) [45]. The OPL’s weak reliability is thus a consequence of a complex, local anatomy and not *per se* noise introduced by small changes in the scan window, as with the other layers. The reliability is therefore less dependent on the thickness estimate than on an exact beam placement during measurement. When investigating OPL in clinical or research settings, an optimal OCT scan with exact beam placement is crucial to prevent uncorrectable OPL differences in the raw data. For this reason, the OSCAR-IB quality criteria, particularly criterion B (“beam placement”), should be followed rigorously [46–48].

Our study has the following limitations: data acquisition and manual correction was performed by different operators for the study cohorts, but nevertheless results were still very similar in patients and controls. However, in real multicenter settings more influencing factors are possible, like the use of different scanners or software versions or multiple operators. Further studies are necessary to tackle these issues and such studies would require the inclusion of several operators at different centers, which was beyond the scope our study. Furthermore, we did not have access to analyzable data from RT-Vue and Topcon; and the raw data of these devices could behave differently when looking at different thickness estimates. Lastly, the sample size of our study was relatively low and given its exploratory nature we were not able to perform a-priory sample size calculations. However, CI were very narrow for the excellent thickness estimates and we therefore believe that our data still support the notion of excellent repeatability of some thickness estimates and particular layers.

Several clinical trials using OCT-derived parameters as primary or secondary study outcome parameters are ongoing or are about to commence. Current and future studies should favor the most reliable thickness estimates over weaker ones. Although the thickness estimates A—E all performed well, the overall performance of estimate A–the mean of all values in the 6mm-diameter ring area centered on and including the fovea–was above average and we strongly encourage clinical researchers, as well as device manufacturers, to rely on this thickness estimate when implementing intra-retinal segmentation measurement across all macular layers. This thickness estimate should also serve as the basis for acquiring normative data from healthy controls to aid comparability between different devices and to detect measurements deviant from healthy subjects. If a proven reliable thickness estimate is not viable within the context of an individual study, scientists should explain how they selected the alternative methods and quantify the reliability of these.

## Supporting Information

S1 Data Results of OCT layer thickness estimate calculations. (ZIP)

S1 File Supplementary materials. This file contains overviews of the study cohorts (Table A), the scientific articles and OCT scan settings used for retinal OCT segmentation in patients with multiple sclerosis (Table B) and the applied segmentation methods used in the retrieved articles (Table C).
(DOCX)
