# Supplementary material for: Reliability of Intra-Retinal Layer Thickness Estimates
Source: PLoS One. 2015 Sep 8;10(9):e0137316. doi: 10.1371/journal.pone.0137316 (PMC4562656; doi:10.1371/journal.pone.0137316)
Supplement: S1 File — This file contains overviews of the study cohorts (Table A), the scientific articles and OCT scan settings used for retinal OCT segmentation in patients with multiple sclerosis (Table B) and the applied segmentation methods used in the retrieved articles (Table C). (DOCX) [file pone.0137316.s002.docx]

# S1 File. Supplementary materials.

## Table A: Cohort overview

|  | **Spectralis healthy controls** | **Cirrus healthy controls** | **MS / CIS patients** |
| --- | --- | --- | --- |
| **N patients** | 15 | 15 | 13 (11 MS, 2 CIS) |
| **N eyes (# eyes with history of ON)** | 30 (NA) | 30 (NA) | 26 (10) |
| **Age in years (mean, SD)** | 27.9 (5.5) | 28.3 (4.8) | 42.8 (8.8) |
| **Gender (female:male)** | 6:9 | 8:7 | 6:7 |
| **Disease duration in years (mean, SD) ^a^** | NA | NA | 86.8 (78.3) |

**Abbreviations: ON**: optic neuritis, **NA**: not applicable, **SD**: standard deviation, **MS**: multiple sclerosis, **CIS**: clinically isolated syndrome.

**^a^** only MS patients

## Table B: Overview of scientific articles and OCT scan settings used for retinal OCT segmentation in patients with multiple sclerosis.

| **Reference** | **Date of publication** | **Authors** | **Journal abbreviation** | **OCT device, manufacturer** | **Acquisition software version** | **Scan name / type** | **Scan resolution: #B-scans, #A-scans per B-scan** | **Scan dimension** | **Additional scan settings** |
| --- | --- | --- | --- | --- | --- | --- | --- | --- | --- |
| 1 | 01.05.2008 | Choi et al. | IOVS | Fourier Domain OCT, experimental device | NA | Volume scan | 100 B-scans, 1000 A-scans | 6 x 6 x 2 mm |  |
| 2 | 01.02.2011 | Saidha et al. | Brain | Cirrus 4000, Carl Zeiss Meditec | Version 5.0 | Macular cube protocol | 128 B-scans, 512 A-scans | 6 x 6 x 2 mm |  |
| 3 | 25.05.2011 | Garas et al. | Eur J Ophthalmol | RTVue-100 FD-OCT, Optovue Inc. | Version 4.0 | Standard GCC scan protocol | (1 horizontal B-scan, 934 A-scans and 15 vertical B-scans, 800 A-scans) | (7 × 7 mm) |  |
| 4 | 01.09.2011 | Davies et al. | J Neuroophthalmol | Spectralis OCT, Heidelberg Engineering | NA | Fast macular scan | 25 B-scans, (512 A-scans) | 20° x 20° | ART = 9 frames |
| 5 | 01.12.2011 | Saidha et al. | Mult Scler | Cirrus 4000, Carl Zeiss Meditec | Version 5.0 | Macular cube protocol | 128 B-scans, 512 A-scans | 6 x 6 x 2 mm |  |
| 6 | 26.01.2012 | Tátrai et al. | PLoS ONE | Stratus OCT, Carl Zeiss Meditec | NA | Macular thickness map protocol | 6 B-Scans, (128 A-scans) | 6-mm-diameter disc |  |
| 7 | 01.02.2012 | Syc et al. | Brain | Cirrus HD-OCT, Carl Zeiss Meditec | Version 5.0 | Macular cube protocol | 128 B-scans, 512 A-scans | 6 x 6 x 2 mm |  |
| 8 | 01.03.2012 | Sriram et al. | IOVS | Spectralis HRA + OCT, Heidelberg Engineering | NA | Radial scan protocol | 6 B-scans, 1536 A-scans | 30° of visual angle | ART = 100 frames |
| 9 | 01.06.2012 | Walter et al. | Ophthalmology | Cirrus HD-OCT, Carl Zeiss Meditec | NA | Macular cube protocol | 200 B-scans, 200 A-scans or 128 B-scans, 512 A-scans | 6 x 6 x 2 mm |  |
| 10 | 01.10.2012 | Seigo et al. | J. Neurol | Spectralis OCT, Heidelberg Engineering | Version 5.2.4 | Fast macular protocol | (25 B-scans, 512 A-scans) | (20° x 20°) | ART = 16 |
|  |  |  |  | Cirrus HD-OCT 4000,Carl Zeiss Meditec | Version 5.0 | Macular cube protocol | 128 B-scans, 512 A-scans | 6 x 6 x 2 mm |  |
| 11 | 01.10.2012 | Albrecht et al. | Mult Scler | Spectralis OCT, Heidelberg Engineering | NA | Single horizontal foveal scan | 1 B-scan, (1536 A-scans) | (30°) | ART = 100 frames |
| 12 | 01.11.2012 | Saidha et al. | Lancet Neurol | Cirrus 4000, Carl Zeiss Meditec | Version 5.0 | Macular cube protocol | 128 B-scans, 512 A-scans | 6 x 6 x 2 mm |  |
| 13 | 01.01.2013 | Ratchford et al. | Neurology | Cirrus 4000, Carl Zeiss Meditec | Version 5.0 | Macular cube protocol | 128 B-scans, 512 A-scans | 6 x 6 x 2 mm |  |
| 14 | 01.01.2013 | Sotirchos et al. | Curr Eye Res | Spectralis OCT, Heidelberg Engineering | (Software version 5.2.4) | (Fast macular protocol) | (25 B-scans, 512 A-scans) | (20° x 20°) | (ART = 16) |
|  |  |  |  | Cirrus HD-OCT, Carl Zeiss Meditec | (Version 5.0) | (Macular cube protocol) | (128 B-scans, 512 A-scans) | (6 x 6 x 2 mm) |  |
| 15 | 01.01.2013 | Saidha et al. | JAMA Neurol | Cirrus 4000, Carl Zeiss Meditec | Version 5.0 | Macular cube protocol | 128 B-scans, 512 A-scans | 6 x 6 x 2 mm |  |
| 16 | 01.02.2013 | Fernandes et al. | Ophthalmology | 3D OCT-1000, Topcon Corp. | NA | Macular volume | 128 B-scans, 512 A-scans | 6-mm area |  |
| 17 | 01.04.2013 | Zimmermann et al. | Mult Scler | Cirrus HD-OCT, Carl Zeiss Meditec | Version 5.1 | Macular Cube | 200 B-scans, 200 A-scans or 128 B-scans, 512 A-scans | 6 x 6 x 2 mm |  |
| 18 | 09.04.2013 | Sotirchos et al. | Neurology | Cirrus 4000, Carl Zeiss Meditec | Version 5.0 | Macular cube protocol | 128 B-scans, 512 A-scans | 6 x 6 x 2 mm |  |
| 19 | 21.06.2013 | Schneider et al. | PLoS ONE | Spectralis OCT, Heidelberg Engineering | NA | Custom macular volume scan | 61 B-scans, 786 A-scans | 25° x 30° | ART = 13 frames |
| 20 | 01.07.2013 | Gabilondo et al. | J Neurol Neurosurg Psychiatry | Spectralis OCT, Heidelberg Engineering | NA | Macular raster scan | 25 B-scans, NA | 20° x 20° |  |
| 21 | 06.08.2013 | Kaufhold et al. | PLoS ONE | Spectralis OCT, Heidelberg Engineering | Eye Explorer viewing module versions 5.2.4.0 to 5.6.1.0 | Custom macular volume scan | 61 B-scans, 786 A-scans | 25° x 30° | ART = 13 frames |
| 22 | 03.10.2013 | Kaushik et al. | PLoS ONE | Spectralis HRA + OCT, Heidelberg Engineering | NA | Radial scan protocol | 6 B-scans, 1536 A-scans | 30° of visual angle | ART = 100 frames |
| 23 | 01.12.2013 | Oberwahrenbrock et al. | Mult Scler | Spectralis OCT, Heidelberg Engineering | NA | Custom macular volume scan | 61 B-scans, 786 A-scans | 25° x 30° | ART = 13 frames |
| 24 | 15.12.2013 | Tugcu et al. | J Neurol Sci | RTVue-100 FD-OCT, Optovue Inc. | Version 3.0 | Ganglion Cell Complex (GCC) scan protocol | (1 horizontal B-scan, 934 A-scans and 15 vertical B-scans, 800 A-scans) | (7 × 7 mm) |  |
| 25 | 08.01.2014 | Balk et al. | Mult Scler | Spectralis OCT, Heidelberg Engineering | Software version 1.7.1.0 | Macular volume scan | 25 B-scans; NA | 20° x 20° |  |
| 26 | 01.02.2014 | Park et al. | Acta Ophthalmol | Spectralis OCT, Heidelberg Engineering | NA | Horizontal and vertical SD-OCT line scans | (2 B-scans); 512 A-scans | NA |  |
| 27 | 01.02.2014 | Garcia-Martin et al. | Ophthalmology | Spectralis OCT, Heidelberg Engineering | Spectralis software version 3.2 | Fast macular scan | 25 B-scans, (512 A-scans) | (20° x 20°) | (ART = 9 frames) |

Note: Data in parentheses were not explicitly given in the articles but were presumed from the context (i.e. from the scan name) or deduced from cited references. NA = Not available.

## Table C: Overview of the applied segmentation methods used in the retrieved articles.

| **Method** | **Area of analysis / Point estimate** | **Type** | **Used in reference(s)** | **Segmentation software / version** | **Retinal layers** | | | | | | | | | **Additional combined layers** |
| --- | --- | --- | --- | --- | --- | --- | --- | --- | --- | --- | --- | --- | --- | --- |
|  |  |  |  |  | NFL | GCL | IPL | INL | OPL | ONL | IS | OS | RPE |  |
|  | Annulus with inner radius 0.54 mm and outer radius 2.4 mm | Automatic | 2, 7 | Cirrus software / NA |  | x | | x | | x | | |  | RNFL+GCL+IPL |
|  |  |  | 5, 12, 14 | Cirrus software / NA |  | x | | x | | x | | |  |  |
| K, L |  |  | 10, 15, 18 | Cirrus software / NA | x | x | | x | | x | | |  |  |
|  |  |  | 13 | Cirrus software / NA |  | x | |  |  |  |  |  |  |  |
|  |  |  | 17 | Cirrus software / version 6.0 |  | x | |  |  |  |  |  |  |  |
|  | Global average thickness was computed for each layer within the 6-mm diameter centered at the foveola. Average thicknesses were also calculated for each of the macular areas: A central 1-mm disc (representing the foveal area), as well as inner and outer rings (from 2 to 3 mm and 3 to 6 mm, respectively), subdivided into superior, nasal, inferior, and temporal quadrants. | Automatic | 9 | Custom algorithm | x | x | | x | | x | | | | RNFL+GCL+IPL |
| A | ("… Spectralis software estimating the total GCL volume from the cumulative frames integrated over 25 sections") | Manual | 4 | Spectralis software / NA |  | x |  |  |  |  |  |  |  |  |
| G | "The thickness of each layer was measured at seven points for each hemifield, which were equally distributed between 1.75° and 7° of eccentricity." (Only the vertical B-scan through the fovea was used for segmentation) | Automatic | 8 | Custom algorithm |  | x | | x |  |  |  |  |  |  |
| H | The central horizontal macular B-scan (traversing the fovea); macular width of 5.0 mm | Semiautomatic and manual | 10 | MATLABv7.4 (Mathworks) and Spectralis software | x | x | | x |  | x | x | x | | INL+OPL, ONL+IS+OS |
|  |  | Manual | 14 | (Spectralis OCT, software version 5.2.4) |  | x | | x | x | x |  |  |  | INL+OPL, ONL+IS+OS |
| J | "measured at the thickest point nasally and temporally of the macula"; ONL was measured at the central scan position | Manual | 11 | Heidelberg Eye Explorer software |  | x | | x | x | x |  |  |  |  |
|  |  |  | 14 | (Spectralis OCT, software version 5.2.4) |  | x | | x | x | x |  |  |  | INL+OPL, ONL+IS+OS |
| E | "… on the central B-scan through the fovea and on three B-scans each in nasal and temporal direction."; "… within a box centered around the fovea and excluding the superior and inferior parts ..."; | Semiautomatic | 19 | Spectralis beta software (Version 5.5.0.5, Eye Explorer Software 1.7.0.0) | x | x | x | x | x | | |  |  |  |
| B | 6-mm ring area with exclusion of the central 1 mm diameter area | Semiautomatic | 20 | Spectralis Viewer software, V.5.7, with Segmentation Editor (β version) | x | x | | x | | | | | |  |
| C | "central B-scan through the fovea and every fourth B-scan in temporal and nasal direction" | Semiautomatic | 21 | Heidelberg Software 1.7.1.0 | x | x | | x |  |  |  |  |  |  |
|  |  |  | 23 | Heidelberg Engingeering Sofware; Segmentationseditor (Beta-Version) | x | x | x | x |  |  |  |  |  | GCL+IPL |
| D | "The thickness of each layer was calculated at 3 separate points along each radial line. Given that there were 12 radial lines, a total of 36 points around the macula were measured." | Automatic | 22 | Custom designed algorithm | x | x | | x |  |  |  |  |  |  |
| F | perimacular rim with 3 mm diameter | Semiautomatic | 25 | Software Version 1.7.1.0. | x | x | | x | x | | | | |  |
| I | mean values at 9 locations: mean value obtained at the foveal centre from the horizontal and vertical scans; 490 - 500 µm and 1490 - 1500 µm from the fovea in temporal, nasal, superior, inferior direction, respectivly. | Manual | 26 | manually measured using the calipers provided with the SD-OCT instrument | x | x | | x | x | x | x | x | x |  |
|  | 666 mm2 | Automatic | 27 | "new segmentation application (Segmentation Technology; Heidelberg Engineering, Inc.)" | Retinal layer assignment seems incorrect and mean thickness values are not in accordance to retinal anatomy. Please see our letter to the authors.^[[1]](#footnote-1)^ | | | | | | | | | |
|  | NA | Semiautomatic | 1 | NA | x | | |  |  |  |  |  |  |  |
|  | "...the area above and below the horizontal meridian", "one horizontal line with 7 mm scan length, followed by 15 vertical lines with 6 mm scan length and 0.5 mm interval, centered 1 mm temporal to fovea" | Automatic | 3 | RTVue-100 software / version 4.0 | x | | |  |  |  |  |  |  |  |
|  |  |  | 24 | RTVue-100 software / version 3.0 | x | | |  |  |  |  |  |  |  |
|  | "… nine macular regions defined by the Early Treatment Diabetic Retinopathy Study (ETDRS)" | Semiautomatic | 6 | OCTRIMA / NA | x | x | | x | x | x | |  | x | RNFL+GCL+IPL |
|  | (6 x 6 mm area) | Semiautomatic | 16 | "validated automated segmentation algorithm"; (MatLab) | x | x | | x |  |  |  |  |  |  |

## References

1. Choi SS, Zawadzki RJ, Keltner JL, Werner JS. Changes in Cellular Structures Revealed by Ultra-high Resolution Retinal Imaging in Optic Neuropathies. Invest Ophthalmol Vis Sci. 2008;49(5):2103-2119. doi:10.1167/iovs.07-0980.

2. Saidha S, Syc SB, Ibrahim MA, et al. Primary retinal pathology in multiple sclerosis as detected by optical coherence tomography. Brain. 2011;134(2):518-533. doi:10.1093/brain/awq346.

3. Garas A, Simó M, Holló G. Nerve fiber layer and macular thinning measured with different imaging methods during the course of acute optic neuritis. Eur J Ophthalmol. 2011;21(4):473-483. doi:10.5301/EJO.2010.5844.

4. Davies EC, Galetta KM, Sackel DJ, et al. Retinal Ganglion Cell Layer Volumetric Assessment by Spectral-Domain Optical Coherence Tomography in Multiple Sclerosis: Application of a High-Precision Manual Estimation Technique. J Neuro-Ophthalmol Sept 2011. 2011;31(3):260-264. doi:10.1097/WNO.0b013e318221b434.

5. Saidha S, Syc SB, Durbin MK, et al. Visual dysfunction in multiple sclerosis correlates better with optical coherence tomography derived estimates of macular ganglion cell layer thickness than peripapillary retinal nerve fiber layer thickness. Mult Scler J. 2011;17(12):1449-1463. doi:10.1177/1352458511418630.

6. Tátrai E, Simó M, Iljicsov A, Németh J, DeBuc DC, Somfai GM. In Vivo Evaluation of Retinal Neurodegeneration in Patients with Multiple Sclerosis. PLoS ONE. 2012;7(1):e30922. doi:10.1371/journal.pone.0030922.

7. Syc SB, Saidha S, Newsome SD, et al. Optical coherence tomography segmentation reveals ganglion cell layer pathology after optic neuritis. Brain. 2012;135(2):521-533. doi:10.1093/brain/awr264.

8. Sriram P, Graham SL, Wang C, Yiannikas C, Garrick R, Klistorner A. Transsynaptic Retinal Degeneration in Optic Neuropathies: Optical Coherence Tomography Study. Invest Ophthalmol Vis Sci. 2012;53(3):1271-1275. doi:10.1167/iovs.11-8732.

9. Walter SD, Ishikawa H, Galetta KM, et al. Ganglion Cell Loss in Relation to Visual Disability in Multiple Sclerosis. Ophthalmology. 2012;119(6):1250-1257. doi:10.1016/j.ophtha.2011.11.032.

10. Seigo MA, Sotirchos ES, Newsome S, et al. In vivo assessment of retinal neuronal layers in multiple sclerosis with manual and automated optical coherence tomography segmentation techniques. J Neurol. 2012;259(10):2119-2130. doi:10.1007/s00415-012-6466-x.

11. Albrecht P, Ringelstein M, Müller AK, et al. Degeneration of retinal layers in multiple sclerosis subtypes quantified by optical coherence tomography. Mult Scler J. 2012;18(10):1422-1429. doi:10.1177/1352458512439237.

12. Saidha S, Sotirchos ES, Ibrahim MA, et al. Microcystic macular oedema, thickness of the inner nuclear layer of the retina, and disease characteristics in multiple sclerosis: a retrospective study. Lancet Neurol. 2012;11(11):963-972. doi:10.1016/S1474-4422(12)70213-2.

13. Ratchford JN, Saidha S, Sotirchos ES, et al. Active MS is associated with accelerated retinal ganglion cell/inner plexiform layer thinning. Neurology. 2013;80(1):47-54. doi:10.1212/WNL.0b013e31827b1a1c.

14. Sotirchos ES, Seigo MA, Calabresi PA, Saidha S. Comparison of Point Estimates and Average Thicknesses of Retinal Layers Measured Using Manual Optical Coherence Tomography Segmentation for Quantification of Retinal Neurodegeneration in Multiple Sclerosis. Curr Eye Res. 2012;38(1):224-228. doi:10.3109/02713683.2012.722243.

15. Saidha S, Sotirchos ES, Oh J, et al. RElationships between retinal axonal and neuronal measures and global central nervous system pathology in multiple sclerosis. JAMA Neurol. 2013;70(1):34-43. doi:10.1001/jamaneurol.2013.573.

16. Fernandes DB, Raza AS, Nogueira RGF, et al. Evaluation of Inner Retinal Layers in Patients with Multiple Sclerosis or Neuromyelitis Optica Using Optical Coherence Tomography. Ophthalmology. 2013;120(2):387-394. doi:10.1016/j.ophtha.2012.07.066.

17. Zimmermann H, Freing A, Kaufhold F, et al. Optic neuritis interferes with optical coherence tomography and magnetic resonance imaging correlations. Mult Scler J. 2013;19(4):443-450. doi:10.1177/1352458512457844.

18. Sotirchos ES, Saidha S, Byraiah G, et al. In vivo identification of morphologic retinal abnormalities in neuromyelitis optica. Neurology. 2013;80(15):1406-1414. doi:10.1212/WNL.0b013e31828c2f7a.

19. Schneider E, Zimmermann H, Oberwahrenbrock T, et al. Optical Coherence Tomography Reveals Distinct Patterns of Retinal Damage in Neuromyelitis Optica and Multiple Sclerosis. PLoS ONE. 2013;8(6):e66151. doi:10.1371/journal.pone.0066151.

20. Gabilondo I, Sepúlveda M, Ortiz-Perez S, et al. Retrograde retinal damage after acute optic tract lesion in MS. J Neurol Neurosurg Psychiatry. 2013;84(7):824-826. doi:10.1136/jnnp-2012-304854.

21. Kaufhold F, Zimmermann H, Schneider E, et al. Optic Neuritis Is Associated with Inner Nuclear Layer Thickening and Microcystic Macular Edema Independently of Multiple Sclerosis. PLoS ONE. 2013;8(8):e71145. doi:10.1371/journal.pone.0071145.

22. Kaushik M, Wang CY, Barnett MH, et al. Inner Nuclear Layer Thickening Is Inversley Proportional to Retinal Ganglion Cell Loss in Optic Neuritis. PLoS ONE. 2013;8(10):e78341. doi:10.1371/journal.pone.0078341.

23. Oberwahrenbrock T, Ringelstein M, Jentschke S, et al. Retinal ganglion cell and inner plexiform layer thinning in clinically isolated syndrome. Mult Scler J. 2013;19(14):1887-1895. doi:10.1177/1352458513489757.

24. Tugcu B, Soysal A, Kılıc M, et al. Assessment of structural and functıonal vısual outcomes ın relapsıng remıttıng multıple sclerosıs wıth vısual evoked potentıals and optıcal coherence tomography. J Neurol Sci. 2013;335(1–2):182-185. doi:10.1016/j.jns.2013.09.027.

25. Balk LJ, Tewarie P, Killestein J, Polman CH, Uitdehaag BMJ, Petzold A. Disease course heterogeneity and OCT in multiple sclerosis. Mult Scler J. 2014:1352458513518626. doi:10.1177/1352458513518626.

26. Park K-A, Kim J, Oh SY. Analysis of spectral domain optical coherence tomography measurements in optic neuritis: differences in neuromyelitis optica, multiple sclerosis, isolated optic neuritis and normal healthy controls. Acta Ophthalmol (Copenh). 2014;92(1):e57-e65. doi:10.1111/aos.12215.

27. Garcia-Martin E, Polo V, Larrosa JM, et al. Retinal Layer Segmentation in Patients with Multiple Sclerosis Using Spectral Domain Optical Coherence Tomography. Ophthalmology. 2014;121(2):573-579. doi:10.1016/j.ophtha.2013.09.035.

1. Re: Garcia-Martin et al.: Retinal layer segmentation in patients with multiple sclerosis using spectral domain optical coherence tomography (Ophthalmology 2014;121:573–9)

   Oberwahrenbrock, Timm et al.

   Ophthalmology , Volume 121 , Issue 11 , e63

   <http://www.aaojournal.org/article/S0161-6420%2814%2900531-4/fulltext> [↑](#footnote-ref-1)
